# A Single Injection of NTG-101 Reduces the Expression of Pain-Related Neurotrophins in a Canine Model of Degenerative Disc Disease

**DOI:** 10.3390/ijms23105717

**Published:** 2022-05-20

**Authors:** Ajay Matta, Muhammad Zia Karim, Hoda Gerami, Bettina Zoe Benigno, Ivan Cheng, Arne Mehrkens, William Mark Erwin

**Affiliations:** 1 Notogen Inc., Toronto, ON M5G OB7, Canada; amatta@notogen.com (A.M.); zkarim@notogen.com (M.Z.K.); hgerami@notogen.com (H.G.); bbenigno@notogen.com (B.Z.B.); 2 Austin Spine, Austin, TX 78705, USA; ivanchengmd@gmail.com; 3 University of Basel, Spitalstrasse 21, 4031 Basel, Switzerland; arne.mehrkens@usb.ch; 4 University of Toronto, Toronto, ON M5G 0B7, Canada; 5 Canadian Memorial Chiropractic College, Toronto, ON M2H 3J1, Canada

**Keywords:** pain, degenerative disc disease, NGFr, TrkB, BDNF, CALCRL

## Abstract

Background: Tissue sources of pain emanating from degenerative discs remains incompletely understood. Canine intervertebral discs (IVDs) were needle puncture injured, 4-weeks later injected with either phosphate-buffered saline (PBS) or NTG-101, harvested after an additional fourteen weeks and then histologically evaluated for the expression of NGFr, BDNF, TrkB and CALCRL proteins. Quantification was performed using the HALO automated cell-counting scoring platform. Immunohistochemical analysis was also performed on human IVD tissue samples obtained from spinal surgery. Immunohistochemical analysis and quantification of neurotrophins and neuropeptides was performed using an in vivo canine model of degenerative disc disease and human degenerative disc tissue sections. Discs injected with NTG-101 showed significantly lower levels of Nerve Growth Factor receptor (NGFr/TrkA, *p* = 0.0001), BDNF (*p* = 0.009), TrkB (*p* = 0.002) and CALCRL (*p* = 0.008) relative to PBS injections. Human IVD tissue obtained from spinal surgery due to painful DDD show robust expression of NGFr, BDNF, TrkB and CALCRL proteins. A single intradiscal injection of NTG-101 significantly inhibits the expression of NGFr, BDNF, TrkB and CALCRL proteins in degenerative canine IVDs. These results strongly suggest that NTG-101 inhibits the development of neurotrophins that are strongly associated with painful degenerative disc disease and may have profound effects upon the management of patients living with discogenic pain.

## 1. Introduction

Back and neck pain secondary to degenerative disc disease (DDD) is the health condition with the highest economic cost to society, with direct treatment costs alone estimated to be at least USD 2 billion annually in the United States [1,2,3]. Aspects associated with DDD include the loss of disc height, cellularity, structural integrity and biomechanical properties, factors that can summate in the generation of pain, instability and deformity [4]. Since there is no cure, current treatments for DDD are limited to symptom management, such as physical therapies, anti-inflammatory medications, and analgesics. The role of surgery in the treatment of DDD is extremely limited, reserved for cases of deformity, instability, or compression of the neural elements. DDD is a multifactorial condition, but trauma, ageing, genetics, occupational stresses and associated molecular signaling within the IVD lead to a catabolic, pro-inflammatory cascade resulting in progressive tissue damage [4,5]. These extremely limited treatment modalities create an urgent need for a disease modifying intervention. We have previously shown that a single intradiscal injection of NTG-101, a novel drug containing Connective Tissue Growth Factor (CTGF) and Transforming Growth Factor Beta-1 (TGF-β1) within an excipient solution inhibits inflammation induced catabolism, robustly increases anabolic repair, preserves biomechanical properties and disc height [6,7,8].

The generation of pain associated with DDD continues to be the subject of controversy due to the multiplicity of anatomical sites capable of causing pain as well as the complex realities of non-organically mediated pain. With respect to anatomically generated pain, it has been extensively reported that painful intervertebral discs (IVD) strongly express proteins (neurotrophins) that are associated with painful DDD, including nerve growth factor and its receptor (NGFr/TrkA), brain derived neurotrophic factor (BDNF), the BDNF receptor (TrkB), the neuropeptides calcitonin gene related peptide (CGRP) and its receptor (CALCRL), and Substance *p* (Sub *p*) [9,10,11]. Neurotrophins are unique growth and survival factors that serve to regulate neuronal survival, development, function, plasticity, and nociception and help to direct cell fate decisions, axonal growth, dendrite pruning, and synaptic function [11,12,13]. Neurotrophins can function as cytokines that play vital instructional roles influencing a variety of processes including differentiation, chemotaxis and the mediation of inflammatory cell activity [9]. Neurotrophins also play an important role with respect to responses to inflammation, and a positive feedback loop that modulates spinal cord synaptic pathways and associated pain [9].

There are numerous neurotrophins known to exist with the first neurotrophin ‘nerve growth factor’ (NGF) found to be synthesized and secreted by sympathetic and sensory target organs [12]. NGF has been reported to be captured within nerve terminals and transported through axons to neuronal cell bodies where survival and differentiation signaling occurs [12]. In addition to NGF there are other neurotrophins synthesized by sensory neurons such as BDNF [13]. Additionally, sensory neurons also synthesize neuropeptides such as CGRP, and Substance *p* that are associated with pain. For example, in sensory neurons, BDNF is upregulated by stimulation with NGF where it can be transported to both peripheral and central terminals of nociceptive neurons [12]. In fact, BDNF is released when nociceptors are activated and it acts as a central modulator of pain [13]. Treatment of primary sensory neurons with NGF upregulates the release of neuropeptides such as Calcitonin Gene Related Peptide (CGRP)-with its receptor CALRCL, and Substance *p* (Sub *p*) just as NGF induces BDNF expression [13]. NGF also promotes the sprouting of TrkA-expressing nociceptors resulting in hyperinnervation in NGF-target tissues that include the intervertebral disc annulus fibrosus [13]. In the degenerative disc neurotrophin expression is associated with neovascularization and neoinnervation within the IVD that in turn leads to accelerated degradation of the ECM, compromised IVD function, increased sensitization of IVD nociceptors and discogenic pain [14]. Numerous reports concerning low back pain associated with neural ingrowth have reported that neurotrophic factors including NGF, BDNF, TrkA, and TrkB contribute to primary disc pain [14]. It has been shown that pro-inflammatory factors within the degenerative disc such as IL-1β and TNF-α have a stimulatory effect upon the production of NGF and BDNF by IVD cells, providing support for the hypothesis that NGF may contribute to nerve ingrowth and pain generation in degenerative and herniated IVDs. To this end it was first reported by Freemont et al., that non-myelinated nerve fibres penetrated into intervertebral discs presumed to be painful and that these fibres expressed Substance *p* in IVDs associated with pain [15]. Freemont et al., later determined that these unmyelinated nerve fibres that penetrated the IVD expressed TrkA as well as NGF [16]. Yamauchi et al. [17] reported that conditioned medium developed from human IVD tissues obtained from spinal surgery for painful DDD (confirmed from pain reduction following anaesthetic injection into the disc) when cultured with neonatal rat dorsal root ganglia (DRG) cells promoted axonal growth in the cultured DRG cells. In particular, the authors reported that the expression of neuropeptides such as Substance *p* was induced within cultured DRGs only in the presence of media conditoned by degenerative human IVD cells [17]. Collectively this work demonstrated that soluble factors obtained from degenerative human IVD cells promotes the growth of sensory nerve fibres innervating the painful disc and may induce pain associated neuropeptides such as Substance *p* [17]. This work provides support for the hypothesis that the pro-inflammatory, degenerative disc secretes neurotophin-inducing soluble factors.

There are numerous sources of pain that may emanate from the IVD. For example, functional/anatomical causes include loss of disc height that can in turn lead to dorsal ganglion and nerve root compression and neuropathic pain. Neuropathic pain may be associated with DDD and the neurological consequences of IVD pathology but is not in itself ‘discogenic’. Imbalances between excitatory and inhibitory somatosensory signaling, and changes in the way that pain messages are modulated within the central nervous system as well as altered ion channel function can lead to various forms of neuropathic pain [18]. “Discogenic pain” on the other hand involves nociception from the IVD that is conveyed by afferent input through the DRG spinal cord dorsal horn and subsequent spinal and supraspinal pain processing. In the case of DDD related pain, pathological IVD changes when coupled with increased levels of IL-1β, TNFα, IL-6, and IL-8 within the degenerative disc can induce IVD NP cells to synthesize NGF and BDNF and lead to pain of IVD origin [11,19]. A study using rat IVD puncture by Sugiura et al., reported that after IVD puncture and saline injection, the respective DRGs subserving the injured IVDs significantly increased their expression of CGRP and that this increased expression was at least in part mediated by the low affinity nerve growth factor P75NTR [20]. This work strongly indicates the capacity of disc injury (likely associated with increased pro-inflammatory cytokine stimulation) to directly affect neurotrophin expression (in this case, within the respective DRGs) likely impacting pain processing secondary to DDD. Increases in pro-inflammatory cytokine levels such as IL-1β (one of several known factors in the catabolic cascade of events leading to DDD) leads IVD cells (in vivo) to increase their expression of NGF, a key element in the development of increased neurotrophins in painful discs [11,17]. Yamauichi et al. [21]., reported that NGF expressed within the NP promotes the proliferation of sensory nerves that inn ervate the degenerative disc. Further, it has been reported that a number of neurotrophins including TrkA, the NGF and BDNF have been detected within the annulus of the IVD, and in the case of BDNF even within the NP of human degenerative IVDs [21]. In a study of healthy and degenerative disc disease, Purmessur et al., reported increased expression of TrkA and TrkB within the IVD in cases of more severe disease [22]. Therefore, therapies that may suppress the over expression of pain-related neurotrophins within the IVD may mitigate DDD-related pain.

## 2. Results

### In Vivo Expression of Neurotrophins/Pain Associated Proteins

**Figure 1:**
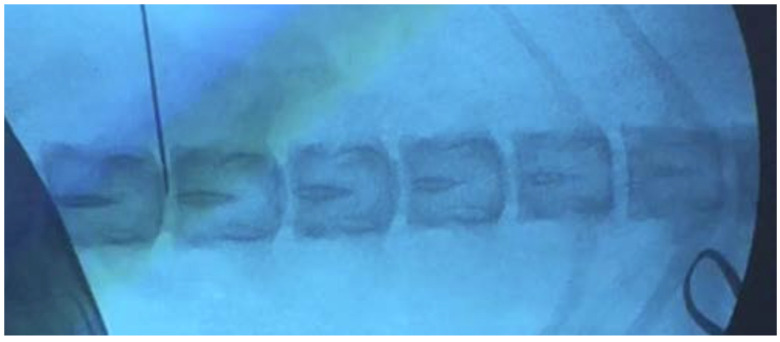
Representative image of fluoroscopically guided 20-gauge needle puncture injury into the L3/4 IVD.

Canine IVDs: We previously showed that a fluoroscopically guided single injection of NTG-101 (Figure 1) preserved the biomechanical properties of the injected discs as compared to saline injected discs [7].

Further, the NTG-101-injected IVDs demonstrated significant increases in aggrecan and collagen 2 expression as well as a suppression of the pro-inflammatory cytokines interleukins -6 and -8 (IL-6 and IL-8) measured at the genomic and protein expression levels [7]. Here, we determined and compared the expression levels of neurotrophins within the annulus fibrosus of canine IVDs injected with saline or NTG-101 following needle puncture as described earlier. We obtained tissue sections from these IVDs and immunostained them for the presence of Nerve Growth Factor receptor (NGFr), Brain Derived Neurotrophic Factor (BDNF), the BDNF receptor (TrkB), and the Calcitonin Gene Related Peptide receptor (CALCRL). Only intra-cellular or membranous staining in AF cells was considered as positive staining in tissue sections.

**Figure 2:**
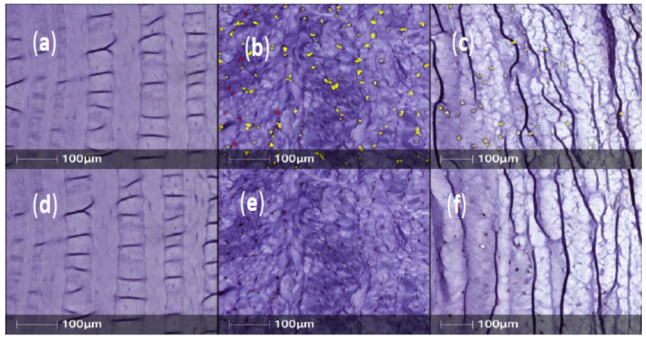
Representative sample of nerve growth factor receptor (NGFr/TrkA) expression in normal, PBS (n = 11) and NTG-101 (n = 16) injected IVDs. (**a**–**c**) Top panels show immunopositively stained cells overlaid in yellow by HALO automated cell counting software. (**d**–**f**) Identical sections without yellow overlay immunostained for TrkA with the DAB chromogen are shown in the bottom panels.

**Figure 3:**
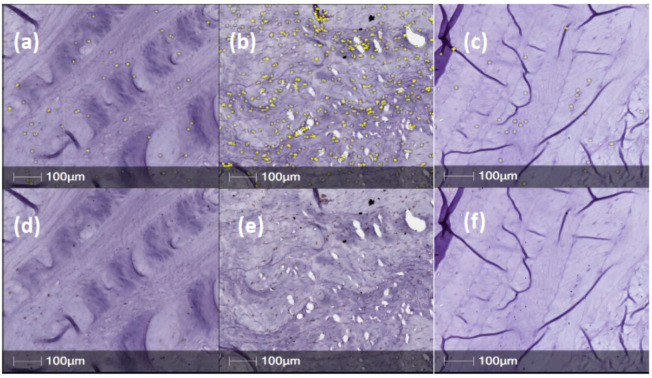
Representative samples for BDNF immunostained canine IVD sections in normal, PBS (n = 7) and NTG-101 (n = 8) injected IVDs. (**a**–**c**) Top. panels show HALO automated cell counting (yellow overlay) over brown DAB chromogen-stained cells for (**a**) normal, (**b**) PBS injected and (**c**) NTG-101 injected discs. (**d**–**f**) Bottom panels are identical images but showing only DAB chromogen immunostained cells.

**Figure 4:**
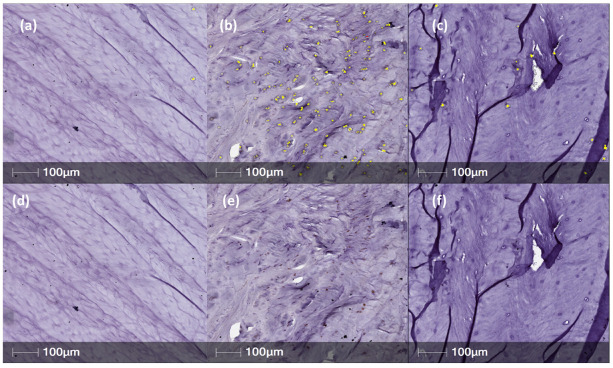
Representative samples for TrkB immunostaining of normal, PBS (n = 11) and NTG-101 (n = 6) injected canine IVDs. (**a**–**c**) Yellow overlay over immunopositive cells as well as only DAB immunopositive cells are shown in top and bottom panels, respectively (**d**–**f**).

**Figure 5:**
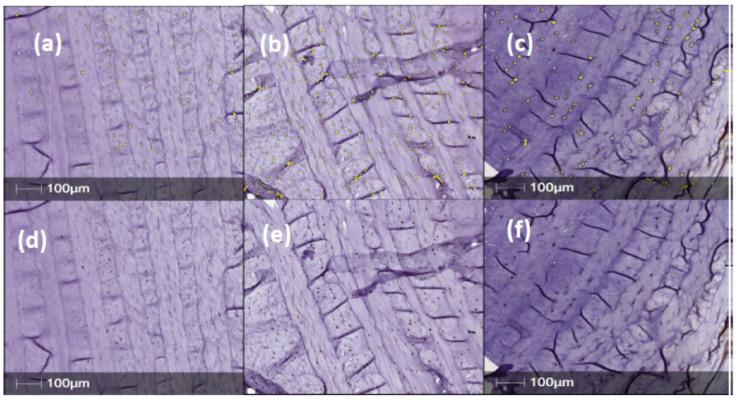
Representative samples for CALCRL immunostaining of canine IVDs in normal, PBS (n = 10) and NTG-101 (n = 12) injected IVDs. (**a**–**c**) Top panels show immunopositive cells with yellow overlay from HALO automated cell counting with bottom panels. (**d**–**f**) showing DAB chromogen immunostained cells.

Annulus fibrosus tissue sections obtained from IVDs injected with PBS (1X) only, showed strong, positive staining for NGFr/TrkA, BDNF, TrkB and CALCRL in posterior/central AF tissue (Figure 2, Figure 3, Figure 4 and Figure 5). However, expression levels of NGFr/TrkA, BDNF, TrkB and CALCRL was low (as compared to PBS injected IVD-AF tissues) or not observed in posterior/central AF tissue of healthy, uninjured IVDs that served as controls or NTG-101 injected IVDs (Figure 2, Figure 3, Figure 4 and Figure 5). No intra-cellular or membranous staining was observed in AF tissue sections used as negative controls wherein the primary antibody was replaced by species specific blocking serum. This differential expression of neurotrophins in degenerative IVD-AF cells is consistent with past reports [23]. Further, we quantified the number of positively stained cells in three distinct ×20 magnification regions of interest (ROI) within the central/posterior annulus fibrosus using the HALO IQ4 software platform as described in Section 4.

**Figure 6:**
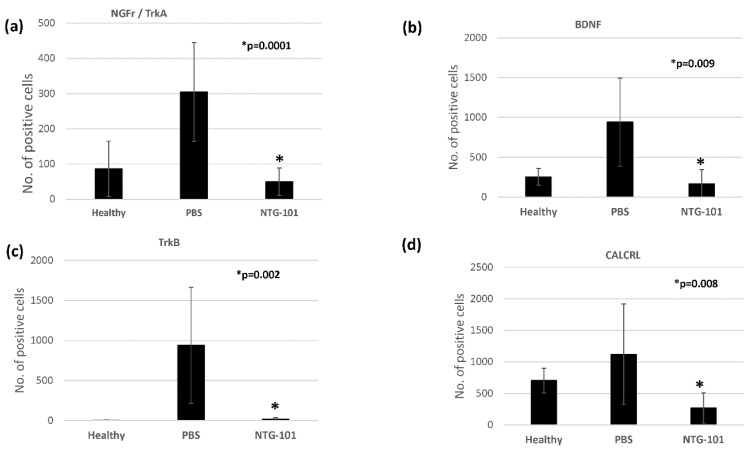
Histograms showing number of quantitative analyses of immunohistochemistry performed for (**a**) NGFr/TrkA, (**b**) BDNF, (**c**) TrkB and (**d**) CALCRL in canine IVD-AF tissue sections. * *p*-values for statistical significance for NTG-101 as compared to PBS-injected canine IVDs.

Statistical analysis was performed using the Student’s t-test for the number of positively stained cells in NTG-101 injected discs and compared to PBS (vehicle) control. HALO IQ4 software revealed that number of AF-cells showing positive immunostaining for NGFr/TrkA, BDNF, TrkB and CALCRL were highest in PBS injected IVD-AF tissue sections as compared to healthy, uninjured control IVDs. However, IVDs that received NTG-101, four weeks post-injury, showed a significantly lower number of positive cells showing immunostaining for NGFr/TrkA (*p* = 0.0001), BDNF (*p* = 0.009), TrkB (*p* = 0.002) and CALCRL (*p* = 0.008) as compared to PBS injected IVD-AF tissue sections (Figure 6).

**Figure 7:**
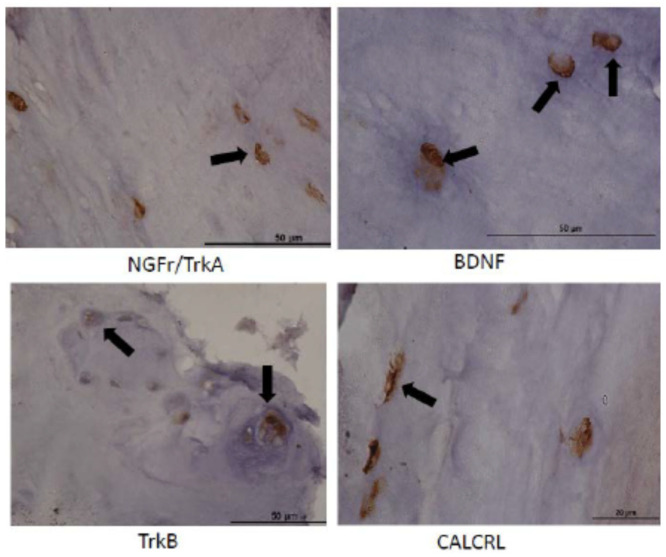
Representative immunostained human IVD tissues stained for NGFr, BDNF, TrkB and CALCRL (magnification × 90 oil immersion). Black arrows depict immunopositive cells for each respective neurotrophins.

Neurotrophin Expression in Human IVDs: We examined human IVD tissues (n = 15) obtained from patients undergoing spinal surgery for discogenic pain as determined by the operative surgeon for the presence of the NGFr, BDNF, TrkB, and CALCRL proteins. Clinical parameters of these patients are given in Table 1. Our cohort included 10 females and 5 males with median age group = 59 years, Pfirrmann grade (range, 2–4) and modic change (range, 0–3). We observed strong expression of NGFr/TrkB (87%), BDNF (80%), TrkB (97%) and CALCRL (100%) cases as shown in Figure 7. Unfortunately, we did not have any healthy human IVD NP samples, therefore there are no comparisons in this study. Nonetheless, we found that these human IVD tissues robustly expressed NGFr, BDNF, TrkB and CALCRL as did the needle puncture injured and saline injected canine IVDs clearly suggesting needle puncture led to the development of degenerative IVD in our canine model of DDD.

## 3. Discussion

The determination of discogenic pain in canine subjects is challenging due to the lack of validated pain measurement tools unlike the case with rodent or feline pain measurement tools such as facial grimacing scales that have been shown to be reliable [24]. Therefore, in this study we examined the differential expression of neurotrophins within needle puncture injured canine IVDs that had variably been injected with PBS or NTG-101 to determine differences in the expression of possible tissue sources of pain in IVDs injected with saline or NTG-101 with respect to untreated controls. Since it has been shown that pro-inflammatory cytokines can induce NGF expression and contribute to the development of DDD [9,22], we examined human spinal surgery IVD tissue samples and probed for the expression of these same neurotrophins.

The nucleus pulposus of degenerative discs is primarily composed of chondrocyte-like cells, and discs obtained from patients undergoing surgery for significant disc-related pain, have been shown to express high levels of interleukins (IL) IL-6 and IL-8 [25]. The healthy IVD is innervated by small diameter sensory neurons that originate from the dorsal root ganglion (DRG) that express NGF, BDNF, Trk A, Trk B and glia cell line-derived neurotrophic factor (GDNF) [9]. Sensory innervation of the non-degenerative human disc is largely restricted to the superficial layers of the annulus fibrosus with the central disc totally lacking in nerve fibres [26,27]. However, in pre-clinical animal models as well as human IVD tissues obtained at surgery for suspected pain due to DDD, have shown that sensory innervation can reach the inner annular layers and even into the nucleus pulposus [15,28]. The IVDs are segmentally innervated although there is some variability in the segmental innervation of the anterior vs. posterior IVD [9,29]. Nerve fibers reach the IVD through the sinuvertebral nerves or from branches of the paravertebral sympathetic trunks [9].

With respect to pain of discogenic origin, it has been reported that neurovascular invasion of the injured/degenerative annulus fibrosus/nucleus pulposus can lead to ingrowth of nociceptive nerves into previously non-innervated IVD tissues as well as the expression of neurotrophins/neuropeptides within these tissues [5,11,15,22]. Indeed, previous reports have shown that the chondrocyte-like cells that populate the IVD NP within painful degenerative discs highly express neurotrophins (plus their receptors) well-known to be involved in the genesis/transmission of nociception such as NGF, TrkA, BDNF, and TrkB and the neuropeptides CGRP and substance *p* [9,10,11,22]. Even though neural ingrowth can occur with degenerative disease, the expression of NGF, TrkA, BDNF, and TrkB by NP cells themselves indicates an independent role played by these neurotrophins apart from neoinnervation. To this end, Sugiura et al., reported in a rat needle puncture model of DDD that after IVD puncture and saline injection, the respective DRGs subserving the injured IVDs significantly increased their expression of CGRP and that this increased expression was at least in part mediated by the low affinity nerve growth factor P75NTR [20]. Furthermore, and in support of the report by Sugiura et al., Krock et al., have shown that proinflammatory cytokines expressed by degenerative discs such as TNF-α and IL-1β are capable of inducing DRG neurons to upregulate expression of the neurotrophins NGF and BDNF and the neuropeptide CGRP (as shown by Sugiura et al.) as well as increased neurite ingrowth [11]. Collectively, this work strongly indicates the capacity of disc injury to directly affect neurotrophin expression (in this case, within the respective DRGs). With respect to the cells within the IVD (AF and NP) much remains to be determined, however an in vitro study by Navone et al., provided evidence that stem cells within the degenerative IVD due to adaptation to an acidic/pro-inflammatory environment are able to develop a neurogenic phenotype that includes the expression of neurotrophin proteins [21]. Collectively these lines of investigation suggest that previously quiescent stem cells become activated secondary to pro-inflammatory stimulation as well as by the anterograde transport of neurotrophic factors from the DRG such as NGF and undergo neurogenic differentiation [21].

We have previously reported that needle puncture injured canine IVDs injected with saline (PBS) undergo significant degeneration whereas a single injection of a novel molecular therapeutic ‘NTG-101′ suppressed degeneration and induced a reparative response [7]. The NTG-101 therapeutic has been developed based upon the necessary and sufficient growth factors identified from the secretome of the notochordal cell-rich IVD found in non-chondrodystrophic canines, animals protected from the development of DDD [6,7]. In fact, notochordal cell-secreted factors have been the subject of considerable interest with respect to extracellular matrix regenerative therapies including a recent review concerning this interesting area of research [30]. Within this study, saline injected canine IVDs led to marked expression of pro-inflammatory cytokines such as IL-1β, TNF-α, IL-6 and IL-8, loss of aggrecan and collagen type 2, impaired biomechanical properties and loss of disc height [7]. However, a single injection of NTG-101 resulted in near normal levels IL-6 and IL-8, upregulated aggrecan and collagen type 2, maintenance of disc height and biomechanical properties to near normal [6,7,8]. The mechanism of action of the NTG-101 therapeutic was shown to include marked anti-inflammatory and anti-catabolic effects upon IVD NP cells by inhibiting the phosphorylation of P38MAPK and NFκB thereby suppressing inflammatory driven extracellular matrix degradation and matrix catabolism [6,7,8]. Furthermore, the growth factors contained within NTG-101 (CTGF and TGF-β1) activate downstream SMAD signaling within IVD NP cells to induce de novo synthesis of extracellular matrix proteins such as aggrecan and collagen type 2. These growth factors further induce increased cell viability and ECM synthesis via phosphorylation of Erk1 and AKT signaling, the results of which assist in the preservation of disc height and biomechanical properties [6,7,8]. IVDs injected with NTG-101 did not develop increased neurotrophin levels significantly differently from untreated control discs in contrast to discs injected with saline strongly indicating that the use of the NTG-101 therapeutic contributed significantly to these results. It is interesting that although we injected either NTG-101 or PBS directly into the IVD NP, there were significant changes in the levels of neurotrophins detected in the annulus fibrosus. It is possible that diffusion of the injectate may be responsible for these effects however this remains to be determined. It is possible that the anti-inflammatory (and possibly pro-anabolic) effects of the NTG-101 therapeutic inhibited the pro-inflammatory driven increase in neurotrophins otherwise seen to develop in discs injected with saline, perhaps in a similar manner to that described in the Sugiura study.

Study Limitations: We were interested in IVD tissue specific effects of the NTG-101 injection and in our previous study did not assess the respective dorsal root ganglia innervating the IVDs for the expression of neurotrophins, therefore we cannot comment on possible changes in these tissues. Furthermore, although increased neurotrophin levels are strongly associated with painful discs, unlike the use of facial grimacing evaluation used in rodents and cats, there is no reliable method with which to quantitatively evaluate pain of discogenic origin in canines. The reasons for this are the great variability in head and face configuration in dogs that leads to difficulty in arriving at uniform measurements.

## 4. Materials and Methods

### 4.1. Induced Degenerative Disc Disease (DDD) in Chondrodystrophic Canines

We previously induced DDD in 3-year-old retired, breeder chondrodystrophic (Beagles, n = 16) canines using image-guided needle puncture injury. Briefly the procedure was as follows:

Three yr-old chondrodystrophic canines were purchased from a licensed animal testing facility (Kingfisher International Inc., Stouffville, Ontario, Canada). After a 2-week acclimatization, image guided needle puncture injury was performed at 3-non-contiguous lumbar IVD-NPs at levels (L1/2, L3/4 and L5/6) by a clinical Veterinarian. All animals recovered uneventfully. Four weeks later, the animals were randomized and under fluoroscopic guidance injected at the same IVD levels on the contralateral side with a single 350.0 µL intra-discal injection of either vehicle (phosphate-buffered saline) (Group 1, n = 6) or NTG-101 (Group 2, n = 10) under fluoroscopic guidance in injured IVDs (Figure 1). The remaining discs adjacent to the injected ones served as no treatment controls (NTCs). 14 weeks post injection at the endpoint (18-weeks post injury) the animals were humanely euthanized, and each lumbar vertebral motion segment was dissected aseptically. From each animal’s lumbar spine, the treated IVDs (either PBS or NTG-101 injected) were removed and one was kept for biomechanical evaluation, one was used for total protein/total RNA, and one was decalcified and fixed for histological evaluation. Intervening no-treatment controls were used in the same way. The IVDs used for biomechanics were snap frozen, subsequently thawed once for testing, then decalcified and fixed for use in the current study. We have previously reported that these interventions resulted in extensively degenerated IVDs that were injured and received PBS injections, whereas the injured discs that received NTG-101 were comparable to no-treatment controls [7]. In the current study, we performed immunohistochemical analysis using tissue sections from paraffin embedded IVDs that were previously analyzed for histological evaluation as well as samples that were used for biomechanical evaluation. We were interested in examining the specimens used for biomechanical testing since the NTG-101 injected discs demonstrated superior biomechanical properties as compared to PBS-injected discs. The notion that these NTG-101-injected discs might also display reduced neurotrophin expression would further support the hypothesis that this kind of molecular therapy may aid in the reduction of discogenic pain.

### 4.2. Tissue Preparation

Paraffin Embedding and Tissue Sections:

Canine Specimens: All canine IVD segments evaluated were either injected with NTG-101 or PBS with additional non-treated segments that served as controls. Some specimens had been previously decalcified and embedded as reported earlier [7]. The additional specimens were sourced from motion segments that had been biomechanically tested. These specimens had been frozen and thawed once for biomechanical testing and then re-frozen. They were then thawed, and prior to embedding all extraneous soft tissues and posterior elements (inclusive of facet joints) were removed from the vertebral bodies. Next, the vertebral bodies were bisected in the axial plane to leave only the superior and inferior 50% of the vertebral bodies as well as the intact intervertebral disc. Next, the specimens were subjected to 10% formalin fixation and decalcified using Cal-Ex^®^-II solution (Fisher Scientific, ON) for 6–8 weeks. Once the IVD tissues were suitably decalcified, they were cut into 2 coronal sections (dorsal and ventral), with a #20 scalpel blade on a #4 handle and then dehydrated using reagent grade alcohol followed by JFC solution (Fisher Scientific, ON). Tissues were paraffinized using the Leica HistoCore Arcadia paraffin embedding station. Histological cassettes containing the tissues were dipped in liquid paraffin and placed within suitable molds to prepare tissue blocks. Once the paraffin/tissue blocks had matured post embedding, the blocks were gross cut using a microtome (Leica) until the vertebra/IVD interface was visible. Next, serial 5 µm-thick tissue sections were cut using a microtome and collected on glass slides and suitably labeled. Next, each 10–15th section was histologically examined using Hematoxylin and Eosin (H&E) staining to determine tissue integrity. Proteoglycan content was determined using Safranin O staining following standard procedures described earlier followed by immunohistochemical staining [7,31].

Human IVD Tissue Sections: We obtained 10% formalin fixed human IVD tissues (n =15) from surgical samples (graciously provided by Dr. Ivan Cheng, Stanford University and Dr. Arne Mehrkens, University of Basel). These disc tissues were obtained at the time of surgery for discogenic pain as diagnosed/determined by the operating surgeon. These tissues were grossly cleaned of debris with a #10 scalpel blade and forceps and copiously washed with phosphate-buffered saline (PBS). Next the fixed tissues fixed using 10% buffered formalin, dehydrated, and embedded in paraffin as described earlier [7]. Once fixed and embedded, the tissues were sectioned (5 µm thick) using a, mounted, and stained for NGFr, BDNF, TrkB and CALCRL proteins using appropriate species-specific antibodies (see Table 2).

#### Immunohistochemistry (IHC)

Following Safranin O staining, serial tissue sections were deparaffinized in xylene followed by hydration in gradient alcohol (100%, 90%, 70% and 50%). Next, the slides were washed in Tris-buffered saline (1X, pH = 7.4) followed by antigen retrieval using a microwave-based heat retrieval method with citrate buffer (0.1 M, pH = 6.0) for 8 min. The slides were allowed to cool to room temperature followed by washing with Tris-buffered saline (TBS, 1X, pH = 7.4, 3 times) containing 0.025% Triton-X-100. Non-specific binding of the primary antibody was blocked using 10% species appropriate serum provided in the Vectastain^tm^ rabbit and mouse kits (Vector Labs, Brockville, ON Canada K6V 5W1. The slides were then incubated with rabbit polyclonal or mouse monoclonal primary antibodies at appropriate dilutions (Table 2) for overnight at +4 °C followed by 3 washes with Tris-buffered saline (TBS-T, 1X, pH = 7.4, containing 0.025% Triton-X-100). The sections were incubated with hydrogen peroxide (0.3% *v*/*v*) for 10 min to block endogenous peroxidase activity, followed by 3 washes with Tris-Buffered Saline and Tween 20 (TBS-T) Tissue sections were incubated with the horse radish peroxidases (HRP)—conjugated goat anti-rabbit or mouse secondary antibody at appropriate dilution for 30 min. Protein expression was detected using diaminobenzidine (DAB) as the chromogen. The sections were counterstained with Meyer’s hematoxylin and mounted with DPX mountant (Millipore-Sigma, ON).

Image Acquisition and Quantitation: These tissue sections were scanned using the Aperio A2 Digital Whole Slide Scanner at the Advanced Optical Microscopy Facility (AOMF), University Health Network. Scanned images were then uploaded on the IQ4 HALO platform (Indica labs, Albuquerque, NM 87114-6374), and the visible immunostained cells within the annulus fibrosis were quantified following suitable algorithm optimization according to the IQ4 user guide. The IQ4 HALO software platform (Halo version 3.0 whole-slide analysis software (Indica Labs, Albuquerque, NM) allows for image quantitation following development of a suitable algorithm to detect the stained cells of interest. Briefly, the tissue classifier module undergoes machine learning to determine the morphological features of the cells of interest and using the IQ4 software interface a classifier was defined using the default Random Forest classifier algorithm. In this case, positively immunostained spindle shaped fibroblasts within the posterior annulus fibrosus were selected for counting. Once appropriate cells were selected the zoom feature filled the voxel box to set the colour of the stained cells. The size of the cells to be counted were then selected after surveying many cells within the region of interest to ensure uniformity of cell section and the *CytoNuclear V1.4* analysis algorithm used to quantify positively stained fibroblasts.

**Figure A1:**
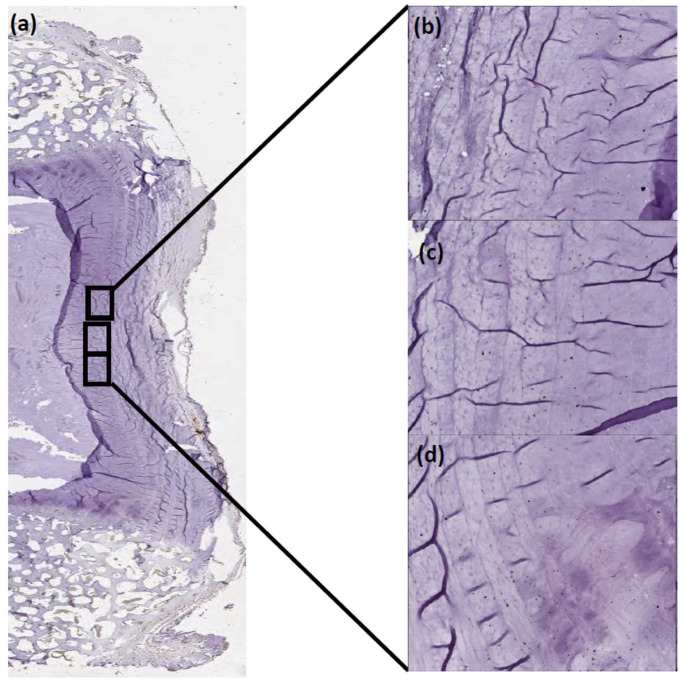
ROI determination for scanned IVD sections. (**a**) The posterior aspect of each scanned tissue section was examined at low power (×2-4) and then at higher power (×20). (**b–d**) Each of three distinct regions at ×20 magnification were selected and immunostained cells were quantified using the Indica Labs IQ4 software programme.

Each scanned glass slide containing a unique IVD tissue section was carefully examined using the Aperio A2 interface and a variety of magnifications from low to high power (x20). We chose to quantitate the immunopositive cells found within the central/posterior aspect of each unique IVD tissue section and investigated regions of interest (ROI) using the HALO software interface to capture as much of the annulus as possible (Figure A1). Once these ROI were outlined, the HALO automated cell counting was performed to capture the immunostained cells within these ROI Automated quantifiable results were obtained for a minimum of three distinct canine IVDs treated with NTG-101, PBS or no treatment controls (NTC) and statistical significance was calculated using the student’s t-test.

## 5. Conclusions

Our results showing suppressed neurotrophin levels after NTG-101 injection compared to saline, when taken together with our earlier work showing the anti-catabolic and pro-anabolic repair effects of NTG-101 [7] suggest that this molecular therapy may play a role in the inhibition of discogenic pain. Further exploration of changes in pain associated with disc disease due to molecular therapy will be important to address in future studies and clinical trials.

## Figures and Tables

**Table 1:** Human patient tissue donor characteristics and expression of neurotrophins (NGFr, BDNF, TrkB and CALCRL) in human degenerative IVD tissue sections.

S. No.	Age (Years)	Gender	Spinal Level	Pfirmann Grade	Modic Type	NGFr *	BDNF *	TrkB *	CALCRL *
1	51	F	L5-S1	III	0	1	1	1	1
2	59	F	L4-L5	II	0	1	1	1	1
3	79	F	L5-S1	IV	0	1	1	1	1
4	67	F	L5-S1	III	1	1	1	1	1
5	57	F	L5-S1	III	1	1	1	1	1
6	66	F	L4–5	IV	0	1	1	1	1
7	78	F	L2–3	III	3	1	1	1	1
8	32	F	L5-S1	IV	1	1	1	0	1
9	30	F	L5-S1	IV	0	1	1	1	1
10	71	M	L5-S1	II	0	1	0	1	1
11	47	M	L5-S1	III	0	1	1	1	1
12	54	F	L4–5	III	0	0	0	1	1
13	78	M	L2–3	V	1	1	1	1	1
14	72	M	L4–5	III	2	0	0	1	1
15	27	M	L4–5	III	0	1	1	1	1

* Immunohistochemical analysis: “1”—positive expression, “0” No detectable expression of protein.

**Table 2:** List of antibodies (source and concentration used) for canine and human IVD sections.

S. No.	IHC Protein	Antibody Source Company	Primary Antibody Cat# and Dilution Used
1.	NGFr/TrkA	R&D Systems	MAB367 (Mouse Monoclonal, 1:50 dilution)
2.	BDNF	Biorbyt	Orb251616 (Rabbit pAb, 1:100 dilution).
3.	TrkB	Biorbyt	Orb214339 (Rabbit pAb, 1:100 dilution)
4.	CALCRL	Antibodies Online	ABIN1048377 (Rabbit Polyclonal, 1:200 dilution)

## Data Availability

Not applicable.

## Abbreviations

BDNF	Brain Derived Nerve Factor
CGRP	Calcitonin Gene Related Peptide
CALCRL	Calcitonin Receptor Like Receptor
CTGF	Connective Tissue Growth Factor
DDD	Degenerative Disc Disease
DRG	Dorsal Root Ganglion
GDNF	Glia Cell Line-Derived Neurotrophic Factor
HRP	Horse Radish Peroxidases
IL-1β	Interleukin- 1 beta
IL-6	Interleukin-6
IL-8	Interleukin-8
IVD	Intervertebral Disc
NGFr	Nerve Growth Factor receptor
NTC	No Treatment Controls
NFkB	Nuclear Factor Kappa B
PBS	Phosphate Buffered Saline
P38MAPK	P38 Mitogen Activated Protein Kinase
TGF-β1	Transforming Growth Factor Beta-1
TBS-T	Tris-Buffered Saline and Tween 20
TrkA	Tyrosine Kinase Receptor A
TrkB	Tyrosine Kinase Receptor B
TNF-α	Tumor Necrosis Factor alpha
rh	Recombinant Human
ROI	Regions of Interest
